# Clinicopathological analysis in patients with neuroendocrine tumors that metastasized to the brain

**DOI:** 10.1186/s12885-015-1999-x

**Published:** 2016-01-22

**Authors:** Jiro Akimoto, Hirokazu Fukuhara, Tomohiro Suda, Kenta Nagai, Megumi Ichikawa, Shinjiro Fukami, Michihiro Kohno, Jun Matsubayashi, Toshitaka Nagao

**Affiliations:** Department of Neurosurgery, Tokyo Medical University, 6-7-1 Nishishinjuku, Shinjuku-ku, Tokyo, 160-0023 Japan; Department of Anatomic Pathology, Tokyo Medical University, Tokyo, Japan

**Keywords:** Neuroendocrine tumor, Metastatic brain tumor, Large cell neuroendocrine tumor, Small cell carcinoma, Radiotherapy, Outcomes

## Abstract

**Background:**

A neuroendocrine tumor (NET) can develop anywhere in the body, but is mainly found in the pancreas, gastrointestinal tract, and lungs. This report is a retrospective study of the clinicopathological features of NET patients with brain metastasis whose tissue diagnosis was made at our hospital.

**Methods:**

Patients with brain metastasis evidenced by clinical records and images were accumulated among 302 patients in whom tissue diagnosis of NETs was made at our hospital between 2008 and 2013. In the patients, the primary lesion, pathological classification, pattern of metastasis, details of treatment, and outcomes were analyzed.

**Results:**

Brain metastasis was observed in 31 patients (10.3 %). The primary lesion was in the lungs in 26 patients (83.9 %), and the mammary glands, esophagus, and uterus in 1 patient each. Primary lesions were unknown in 2 patients, including 1 patient in whom NETs were detected in the lymph nodes alone. Pathological classification of the primary lesion was NET Grade 2 (Ki-67: 3 to 20 %) in 3 patients and neuroendocrine carcinoma (NEC, Ki-67: ≥21 %) in 26 patients. The median period from onset of the primary lesion up to diagnosis of brain metastasis was 12.8 months, and the brain lesion preceded brain metastasis in 6 patients. Ten patients had a single metastasis whereas 21 patients had multiple metastases, but no characteristics were observed in their images. Brain metastasis was extirpated in 10 patients. Stereotactic radiotherapy alone was administered in 6 patients, and brain metastasis was favorably controlled in most of the patients with coadministration of cranial irradiation as appropriate. The median survival period from diagnosis of brain metastasis was 8.1 months, and the major cause of death was aggravation of the primary lesion or metastatic lesions in other organs.

**Conclusion:**

Most of NET patients with brain metastasis showed the primary lesion of NEC in the lungs, and they had multiple metastases to the liver, lymph nodes, bones, and so forth at the time of diagnosis of brain metastasis. The guidelines for accurate diagnosis and treatment of NETs should be immediately established based on further analyses of NET patients with brain metastasis.

## Background

A neuroendocrine tumor (NET) is characterized by production and secretion of peptide hormones, amines, or the presence of secretory granules. The term “carcinoid” was used for a long time because patients with a primary lesion in the gastrointestinal tract were mainly examined and the course was relatively favorable [1].

However, recent research has shown that NETs are not limited to the gastrointestinal tract and originate from dispersed endocrine cells that are widely distributed all over the body. They develop in all organs, and there are not only patients who present a favorable clinical pathology, but many of them also have a malignant course. The World Health Organization (WHO) therefore attempted to grade NETs based on systematic clinical pathological classification, but classifications with different criteria were proposed for NETs originating from the favored sites (pancreas and gastrointestinal tract) and NETs originating from the lungs. Discussions are still continuing concerning the validity of each classification and establishment of standard criteria, but a standard pathological entity is not yet available.

With advances in pathological diagnosis of NETs, neurosurgeons are increasingly experiencing patients with brain metastasis from tumors diagnosed as NETs [2–7]. However, few reports examined brain metastasis of NET patients [2] and therefore, guidelines should be prepared for oncologists based on findings from clinical images and pathology as well as treatment and outcomes of brain metastasis of NETs, and then taking appropriate measures.

We accumulated cases of NET patients with brain metastasis at our hospital and performed clinical pathological analysis. The results are reported below.

## Methods

The terms related to NETs, including “carcinoid”, “endocrine”, “neuroendocrine”, “small cell carcinoma” and “large cell neuroendocrine carcinoma (LCNEC)”, were searched among histopathological diagnoses of all neoplasm specimens obtained during surgical procedures at Tokyo Medical University Hospital during the 6 years between January 2008 and December 2013. As a result, 302 patients were confirmed carrying a diagnosis of NETs. The diagnoses consisted of carcinoid in 75 patients, endocrine in 47 patients, neuroendocrine in 149 patients, small cell carcinoma in 30 patients, and LCNEC in 26 patients. They also included duplications of search terms, and final diagnosis of LCNEC or small cell carcinoma following the initial diagnosis of carcinoma with neuro-endocrine differentiation. The age of patients ranged between 21 and 91 years (median: 64.5 years old), and there were 182 men and 120 women. The primary site of NETs was the lungs in 103 patients, rectum in 37 patients, pancreas in 26 patients, stomach in 20 patients, mammary glands in 20 patients, uterus in 18 patients, lymph nodes in 17 patients, and duodenum in 12 patients. Other sites of onset were the thymus gland in 8 patients, esophagus in 6 patients, ileum in 5 patients, liver in 5 patients, colon in 3 patients, pharynx in 3 patients, paranasal sinus in 3 patients, kidneys in 2 patients, urinary tract in 2 patients, and ovaries in 2 patients.

We then searched medical charts and imaging findings of the 302 patients carrying a diagnosis of NETs and extracted 31 patients (10.3 %) in whom brain metastasis was diagnosed based on either medical charts or imaging findings. In the 31 patients, their clinical images, primary lesion, pathological diagnosis, imaging findings, treatment, and outcomes were retrospectively analyzed.

This study was conducted in accordance with the Helsinki Declaration and was approved by the Ethical Review Board of Tokyo Medical University Hospital. Each patient signed a written informed consent form that was approved by our institutional Committee on Human Rights in Research for the publication of their data.

## Results (Table 1)

### Clinical features

The age of the 31 patients ranged between 34 and 78 years (median: 68 years old) and 19 of them were men and 12 of them were women.

**Table 1:** Clinical pathological images of a patient with brain metastasis originating from NETs confirmed by pathological diagnosis

		N (cases)
Neuroendocrine tumor (NET)		302
Brain metastasis		31 (10.3 %)
Age		34-78 (Median: 68)
Gender		
	Male	19
	Female	12
Origin		
	Lung	26 (83.9 %)
	Breast	1
	Esophagus	1
	Lymph node	1
	Uterus	1
	Unknown	1
Pathology		
	LCNEC	11 (35.5 %)
	Small cell carcinoma	12 (38.7 %)
	Carcinoma with neuroendocrine differentiation	8 (25.8 %)
Grade (Ki-67)		
	2 (3-20 %)	3
	3 (≥21 %)	28 (90.3 %)
Imaging		
	Single/nodular type	7
	Single/cystic type	3
	Multiple/nodular type	10
	Multiple/cystic type	3
	Multiple/nodular and cystic type	8
Treatment		
	Surgery	2
	Surgery + WBRT	7
	Surgery + GK	1
	Surgery + WBRT + GK	1
	WBRT	11
	GK	6
	WBRT + GK	1
	Chemotherapy	1
	Best supportive care	1

*N* number, *LCNEC* large cell neuroendocrine tumor, *WBRT* whole brain radiotherapy, *GK* gamma knife

The primary lesion in 6 patients (19.4 %) was detected by close examination after hospitalization following the onset of intracranial hypertension, disorientation, or local neurological symptoms such as paralysis and cerebellar symptoms, due to brain metastasis. The primary lesion remained unknown in 1 patient in spite of repeated close systemic examinations.

**Fig. 1:**
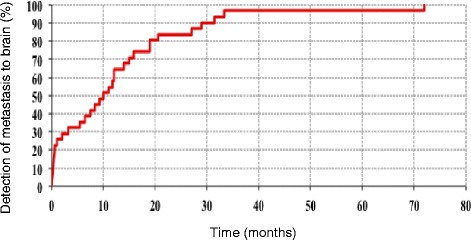
Period from tissue diagnosis of the primary neuroendocrine tumor until diagnosis of brain metastasis in 31 patients

Of the remaining 25 patients in whom brain metastasis was diagnosed during treatment of the primary lesion, brain metastasis was confirmed in 17 patients by imaging, which was performed due to the occurrence of local neurological symptoms or intracranial hypertension, whereas 8 patients did not show definite neurological symptoms and brain metastasis was confirmed by imaging screening. The period from definitive diagnosis of primary lesion up to confirmation of brain metastasis ranged between 0 and 33.4 months (median: 12.8 months) (Fig. 1).

### Primary lesion and pathological diagnosis

The primary lesion was in the lungs in 26 (83.9 %) of 31 patients. Other organs containing the primary lesion included the mammary glands, esophagus, and uterus in 1 patient each, and unknown in 2 patients, including 1 patient in whom NETs were detected in the lymph glands alone. The final pathological diagnosis of lung NETs was large cell neuroendocrine carcinoma (LCNEC) in 10 patients, small cell carcinoma in 10 patients, and carcinoma with neuroendocrine differentiation in 6 patients. The patient with the primary lesion in the mammary glands was diagnosed as having adenocarcinoma with neuroendocrine differentiation, the patient with the primary lesion in the esophagus as having squamous cell carcinoma with neuroendocrine differentiation, and the patient with the primary lesion in the uterus as having LCNEC. A diagnosis of small cell carcinoma was made in the patients with no primary lesion other than in the lymph nodes or with an unknown primary lesion. According to the grading by the percentage of Ki-67 positively stained cells, 3 patients (all of them had NETs originating from the lungs) were Grade 2 (Ki-67: 3 to 20 %) and 28 patients (90.3 %) were Grade 3 (Ki-67: ≥20 %).

### Imaging diagnosis (Fig. 2)

**Fig. 2:**
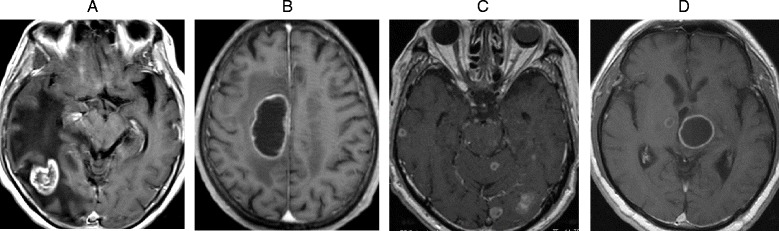
Imaging diagnosis patterns. **a** single/nodular type. **b** single/cystic type. **c** multiple/nodular type. **d** multiple/cystic type

As a result of examination of patterns of metastasis by contrast-enhanced T1-weighted MRI, 7 patients were classified as having the single/nodular type, 3 patients as having the single/cystic type, 10 patients as having the multiple/nodular type, 3 patients as having the multiple/cystic type, and 8 patients as having the multiple/nodular and cystic type. Therefore, no unique imaging patterns characteristic of NET patients with brain metastasis were observed. All the patients demonstrating single brain metastasis had NETs originating from lung, but the patients from non-lung (breast, esophagus, uterus, lymph node) NETs demonstrated multiple brain metastases.

### Treatment

When onset of brain metastasis was confirmed, optimal therapy was chosen and performed based on comprehensive evaluation of the age, performance status (PS), imaging findings, and clinical course of the primary lesion. The lesion that caused symptoms was extirpated as completely as possible in principle, and postoperative cranial irradiation or stereotactic radiotherapy was concomitantly performed as necessary. Details of treatment varied with each patient. For example, the gamma knife was used for a lesion with 3 or fewer small tumors to alleviate symptoms, and cranial radiotherapy alone for a lesion with 4 or more tumors. In addition, treatment was switched to best supportive care at the onset of brain metastasis due to poor PS caused by lesions in other organs. Eventually, the main treatments included craniotomy plus cranial irradiation in 7 patients, cranial irradiation alone in 11 patients, and gamma knife alone in 6 patients.

### Outcomes

**Fig. 3:**
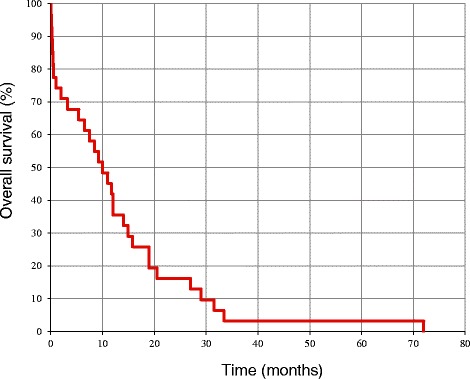
Survival period after diagnosis of metastatic brain tumor

The follow-up period after treatment of metastatic brain tumors was from 1 to 34 months (median: 5.8 months), and 29 of 31 patients were deceased by the time of completion of examinations. Analysis by the Kaplan-Meier survival curve revealed a median survival period of 8.1 months (Fig. 3). Of the 29 deceased patients, only 1 patient died due to central nervous system symptoms caused by uncontrollable brain metastasis. The other patients died because of aggravation of their general condition due to the primary lesion or metastasis to the liver, adrenal gland, lymph nodes, or bones (including the spine), observed at the time of confirmation of brain metastasis. Both of the patients who survived until completion of examinations had the primary lesion in the lungs. Of these 2 patients, 1 patient who was on chemotherapy due to poorly differentiated carcinoma with neuroendocrine differentiation of the single/nodular type was favorably controlled for 34 months with the gamma knife. The other patient who was on chemotherapy for LCNEC developed brain metastasis of the multiple/nodular type, but was favorably controlled for 10 months by the gamma knife.

### Presentation of typical cases

**Fig. 4:**
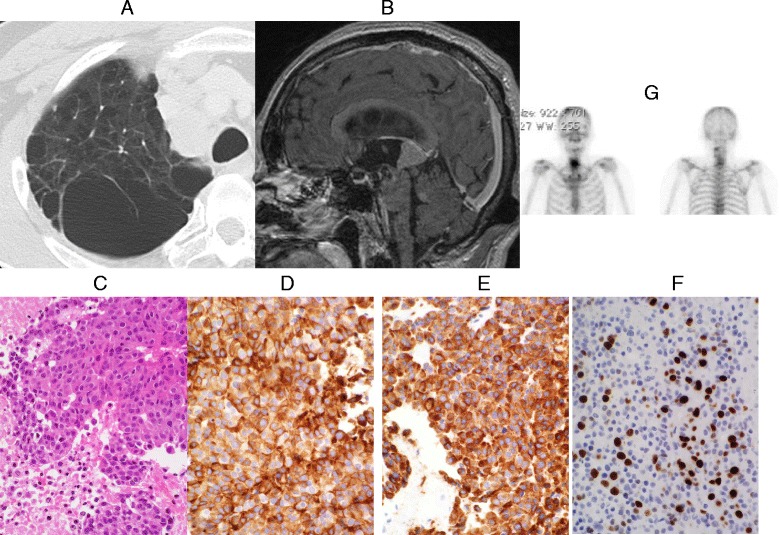
**a** Chest CT scan shows a small lesion (arrow) on the mediastinum at right S2. **b** Sagittal contrast-enhanced T1-weighted head MRI reveals two tumors in the pineal body and the cerebellum accompanied by obstructive hydrocephalus. **c** HE stain image of neuroendoscopic biopsy tissues (original magnification, × 200). **d** anti-synaptophysin antibody. (Rabbit polyclonal, Cell Marque, CA, USA) stain image (original magnification, × 200). **e** anti-chromogranin A antibody (Rabbit polyclonal, Dako, Glostrup, Denmark) stain image (original magnification, × 200). **f** Ki-67 (MIB-1, Dako, Glostrup, Denmark) stain image (original magnification, × 200). **g** Bone scintigraphy shows multiple metastases to the cervical vertebrae
- A patient with no notable medical history and no history of smoking visited our hospital with a complaint of sudden onset of left hemiplegia. Contrast-enhanced head MRI showed multiple tumors with cysts in the right posterior lobe and the parietal lobe (Fig. 5a, b). The tumor in the right parietal lobe was considered to be the cause of the hemiplegia. Therefore, craniotomy was conducted for total resection of the tumor. He received postoperative cranial irradiation at 30 Gy and received ambulatory discharge. Images revealed isolated tissues comprised of pattern-less dense proliferation of small spindle-shaped tumor cells. The cytoplasm of tumor cells was strongly positive for synaptophysin, CD56, TTF1, and CK7 (Fig. 5c-f). Small cell carcinoma originating from the lungs was suspected, although the primary lesion was not identified in spite of examinations by contrast-enhanced whole-body CT scan, gallium scintigraphy, ultrasonography of the thyroid gland and the abdomen, endoscopy of the upper and lower gastrointestinal tract, and whole-body FDG-PET.
  The brain tumors were favorably controlled in the meantime and PS was maintained at 0 or 1 (Fig. 5g, h). However, generalized malaise rapidly increased at around 14 months after brain surgery and the patient died 16 months after surgery while receiving treatment at home. The primary lesion remained unknown.

- A patient with no notable medical history and no history of smoking presented with an abnormal shadow of the chest detected at a medical checkup. A pulmonary CT scan showed a small lesion on the mediastinum at the right S2 (Fig. 4a). The result of transbronchoscopic lung biopsy (TBLB) led to the diagnosis of LCNEC. Contrast-enhanced head MRI was conducted because he presented with headache, diplopia, and disorientation during maintenance chemotherapy with cisplatin and CPT-11 (camptothecin 11). A solid tumor was observed in the pineal body, together with obstructive hydrocephalus. A nodular tumor was observed in the cerebellar parenchyma in the vicinity of the superior medullar velum, which was diagnosed as a metastatic brain tumor of the multiple/nodular type 11.7 months after the pathological diagnosis of the lungs (Fig. 4b). Biopsy for the pineal body tumor and a third ventriculostomy for hydrocephalus were performed by neuroendoscopy. The isolated tissues were comprised of a dense proliferation of large epithelial tumor cells with severe necrotic degeneration, and synaptophysin and chromogranin G were positive and diffused in the cytoplasm. The percentage of Ki-67 positive cells was 35 % and the patient’s lesion was diagnosed as brain metastasis originating from Grade 3 LCNEC (Fig. 4c-f). The patient complained of pain in the posterior cervical region, weakness in the arms, and paresthesia after postoperative cranial irradiation at 30 Gy, and close examination showed metastasis to the cervical vertebra at C3, and the vertebral body and vertebral arch at C6 and C7 (Fig. 4g). Neither the patient nor his family wished to continue treatment. Therefore, the patient was switched to best supportive care at 2.9 months after brain surgery, and died 1 month later due to respiratory failure caused by rapid enlargement of pulmonary lesions.

**Fig. 5:**
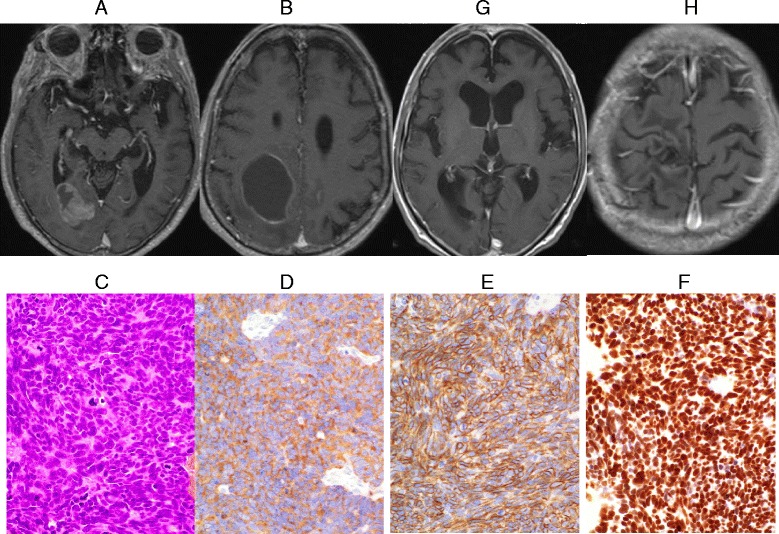
**a**, **b** Horizontal contrast-enhanced T1-weighted head MRI shows multiple tumors with cysts in the right occipital lobe and the parietal lobe. **c** HE image of the isolated parietal lobe tumor tissues (original magnification, × 200). **d** anti-synaptophysin antibody stain image (original magnification, × 200). **e** anti-CD56 antibody (1B6, Leica, Newcastle, UK) stain image (original magnification, × 200). **f** anti-TTF-1 (8G7G3/1, Dako, Glostrup, Denmark) antibody stain image (original magnification, × 200). **g**, **h** Horizontal contrast-enhanced T1-weighted head MRI after cranial irradiation therapy

## Discussion

In 1907, NETs in the small intestine were described as “carcinoid” by Oberndorfer, because the clinical course was relatively favorable and they were pathologically differentiated [1]. Since then, carcinoid has been the description of the disease group for more than 90 years. It was later considered that NETs should be treated as clinically malignant tumors and the term “carcinoid” was eliminated from the WHO classification in 2000. Subsequently, NETs were categorized into five types, namely NET Grade 1, NET Grade 2, NEC (large cell or small cell), mixed adeno-neuroendocrine carcinoma (MANEC), and hyperplastic and neoplastic lesions, in the 2010 WHO classification according to the grading by the Ki-67 labeling index [8]. However, the classification of the pancreatic/gastrointestinal NETs (where the research is the most advanced) is slightly different from that of lung NETs which are sometimes still called “carcinoid”, and the standard pathological classification is yet to be achieved.

NETs are also clinically classified as follows: 1) functional or non-functional NETs, depending on the onset of characteristic hormonal symptoms; 2) foregut origin (lungs/bronchus, thymus, stomach, pancreas, duodenum), midgut origin (jejunum, ileum, appendix), and hindgut origin (colon, rectum), depending on the site of embryological development; 3) whether or not associated with inherited diseases (including multiple endocrine neoplasia type 1, von Hippel-Lindau disease, neurofibromatosis type 1, and tuberous sclerosis) [8]. Therefore, the clinical and pathological definition of NETs is still complicated.

The National Cancer Institute’s Surveillance, Epidemiology, and End Results (SEER) Program (1993 to 2004) searched all registered patients using the International Classification of Disease (ICD) codes such as carcinoid, neuroendocrine carcinoma, and LCNEC, and extracted 17,321 patients with NETs [9]. The incidence of NETs among all tumors was 4.44 % in Caucasians and 6.5 % in African Americans, and male patients were slightly predominant; the incidence increased by 37 % in Caucasians and 40 % in African Americans compared with the statistical data between 1993 and 1997 [9]. The site of onset of NETs in Caucasian patients was the lungs/bronchus in 31.9 %, small intestine in 17.7 %, unknown in 13.5 %, and rectum in 12.3 %, whereas that in African American patients was the rectum in 27 %, small intestine in 21 %, and lungs/bronchus in 18.3 %. Thus, a slight ethnic difference was observed [9]. Also in the percentage of NETs against all types of tumors in each site of origin, NETs accounted for the highest (1.42 %) in the lungs/bronchus in Caucasian patients, whereas NETs accounted for the highest (1.65 %) in the rectum in African American patients [9]. The clinical pathology of pancreatic NETs has been most intensively studied. However, pancreatic NETs accounted for 4 % of all types of the NETs and for approximately 0.3 % of all pancreatic cancers [9], being a very rare disease. Regarding the metastasis of NETs to other organs, distant metastasis was observed in 20 % to 30 % of patients at the time of diagnosis.

Using the search words of SEER [9], we extracted 302 patients with NETs who were diagnosed at our hospital over the past 6 years. The site of onset was the lungs in 103 patients (34.1 %), rectum in 37 patients (12.3 %), and pancreas in 26 patients (8.6 %), which were almost comparable with the site of onset in Caucasian patients, and the number of patients with NETs in the pancreas was higher in Japanese patients. Brain metastasis was observed in 31 patients (10.3 %) and it was interesting that 26 (83.9 %) of them were metastasis from the primary lung NETs. Primary brain metastasis is mainly caused by primary lung NETs whereas pancreatic/gastrointestinal NETs are suggested to reach the brain as the final distal site of metastasis via metastasis to the liver, lymph node, lungs, or other organs.

Brain metastasis from NETs has been frequently reported [2, 4–7] although standardized pathological analysis, diagnosis, and treatment have not been established. The European Neuroendocrine Tumor Society (ENETS) in 2012 published the consensus guidelines for the diagnosis and treatment of neuroendocrine tumors for each site of onset [10]. During preparation of the guidelines, the ENETS advisory board and experts have published the analytical results on metastasis to other organs from NETs [10, 11]. The description of brain, cardiac, and ovarian metastasis in the article stated that brain metastasis from primary lung NETs accounted for 45 % to 71 % of patients with the median period of 1.5 years from the diagnosis of the primary lesion until confirmation of brain metastasis [8]. Lymph node metastasis and liver metastasis were present in 75 % and 50 % of the patients with brain metastasis [11], respectively, which were comparable to the results of our present study. Approximately 1.4 % of metastatic brain tumors were NETs, whereas the incidence of brain metastasis from NETs was 1.5 % to 5 % [11]. The incidence of primary lung NETs was higher in our present study and that would be the cause of slightly higher incidence in brain metastasis at 10.3 %. A report that summarized 24 patients was the highest number of patients and only 57 patients were reported during the 46 years between 1962 and 2007 [11]. Accumulation of 31 patients during 6 years in this study is therefore unsurpassed in terms of analysis of brain metastasis from NETs. The report of ENET [11] did not contain a definite description of pathological findings and grading. Our report may be the first to suggest that most of the patients consist of Grade 3 LCNEC and Grade 3 small cell cancer.

To the best of our knowledge, no report described detailed treatment and outcomes for brain metastasis in NET patients. When limited to lung NETs, brain metastasis occurs in 30 % or more of the LCNEC patients and in 24 % of the small cell carcinoma patients [3, 12]. In the present study, the strategy of surgery and radiotherapy for the patients was determined based on the symptoms, PS, and imaging findings (number of brain metastases), and such action would be the standard concept for metastatic brain tumors at present. On the other hand, prophylactic cranial irradiation is also a possible option for small cell lung cancer [3, 12, 13]. The median survival period from diagnosis of brain metastasis was 8.1 months in the present study, and it was not greatly different from that of brain metastasis in all lung cancer patients [13]. Lately, various therapies such as combination chemotherapy, EGFR tyrosine kinase inhibitors (EGFR-TKI), and angiogenesis inhibitors have become available for brain metastasis originating from lung cancer [13, 14]. Development of optimal therapy is expected in addition to surgery and radiotherapy for brain metastasis originating from NETs based on the accumulation of findings of new chemotherapies and molecular targeted therapies.

## Conclusion

Approximately 10 % of NET patients developed brain metastasis at slightly longer than 1 year after diagnosis, and metastasis originating from LCNEC or small cell carcinoma occurred in most of them. Local recurrence or metastasis, or metastasis to another organ were often present at the time of diagnosis of brain metastasis, and the treatment options for brain metastasis varied depending on the symptoms due to brain metastasis, PS, and metastatic images. Most of the metastases were controlled by applying active therapy to the brain lesion, but the mean survival period was 8 months from diagnosis of brain metastasis, mainly because of aggravation of the primary lesion or metastasis to other organs. Standardization of clinical pathological interpretations of NETs has been attempted in recent years, and such reports are rapidly increasing. Nevertheless, few reports have been published concerning the pathology of brain metastasis of NETs, and the findings of our clinicopathological analysis of this pathology were limited to the experience of our institution. We hope for standardization of clinical and pathological interpretation of NETs and the establishment of optimal guidelines for diagnosis and treatment of brain metastasis originating from NETs by accumulating more information on this disease.

## Abbreviations

ENETS: European Neuroendocrine Tumor Society
ICD: International Classification of Disease
LCNEC: large cell neuroendocrine carcinoma
MANEC: mixed adeno-neuroendocrine carcinoma
NET: neuroendocrine tumor
PS: performance status
SEER: Surveillance, Epidemiology, and End Results
TBLB: transbronchoscopic lung biopsy
WHO: World Health Organization
